# An Improvement of the Efficacy of Moxifloxacin HCl for the Treatment of Bacterial Keratitis by the Formulation of Ocular Mucoadhesive Microspheres

**DOI:** 10.3797/scipharm.1204-08

**Published:** 2012-12-10

**Authors:** Panchaxari Mallappa Dandagi, Amit Manohar Belekar, Vinayak Shivamurthy Mastiholimath, Anand Panchakshari Gadad, Vivek Wamanrao Sontake, Prashant Sanjivrao Salian

**Affiliations:** 1 KLE University’s College of Pharmacy, JNMC Campus, Nehru nagar, 590010, Belgaum, India.; 2 KDCA’s Institute of Pharmacy, Ujalaiwadi, Kolhapur, 416004, India.

**Keywords:** Microspheres, Moxifloxacin HCl, Mucoadhesion, Bacterial keratitis, Ocular

## Abstract

The aim of this study was to prepare novel ocular mucoadhesive microspheres of Moxifloxacin HCl to increase its residence time on the ocular surface and to enhance its therapeutic efficacy in ocular bacterial keratitis. Microspheres were fabricated with different grades of Methocel and Sodium CMC as polymers. Microspheres were evaluated for their particle size, morphology, encapsulation efficiency, mucoadhesion, antimicrobial efficacy, and *in vitro* drug release studies. *In vivo* studies were carried out for the promising formulation on eyes of albino rabbits by inducing bacterial keratitis. A sterile microspheres suspension in light mineral oil was applied to infected eyes twice a day. A marketed conventional eye drop was used as a positive control. Eyes were examined daily for improvement of clinical signs of bacterial keratitis by an ophthalmologist. The average particle size of microspheres was found to be less than 80 μm. Methocel microspheres were found to have a smoother surface than Sodium CMC. Entrapment efficiency was enhanced with an increased polymer concentration and viscosity. The formulation containing Methocel K100M with a drug: polymer ratio of 1:2 exerted longer corneal and conjunctival mucoadhesion time of 8.45±0.15 h and 9.40±0.53 h respectively. *In vitro* release of Moxifloxacin HCl from microspheres was retarded with increased viscosity and concentration of polymers, and was controlled by diffusion as well as polymer relaxation. All formulations showed comparable antimicrobial activity in comparison with conventional marketed eye drops. The formulation containing Methocel K100M with a drug: polymer ratio of 1:2 was found to be a promising formulation and was used for the *in vivo* studies. The *in vivo* studies revealed that microspheres demonstrated significantly lower clinical scores and reduced the total duration of therapy than the marketed Moxifloxacin HCl eye drops. *In vitro* and *in vivo* studies showed that ocular mucoadhesive microspheres of Moxifloxacin HCl were found to have an improved efficacy in the treatment of ocular bacterial keratitis in comparison with the marketed formulation.

## Introduction

Topical application of drugs to the eye is the most popular and well-accepted route of administration for the treatment of various eye disorders. The bioavailability of ophthalmic drugs is very poor due to efficient protective mechanisms of the eye. Blinking, baseline as well as reflex lachrymation and nasolacrimal drainage, removes drug rapidly from the surface of the eye [1, 2]. Whenever a drug is applied topically to the anterior segment of the eye, only a small amount (about 5%) actually penetrates the cornea and reaches the internal anterior tissue of the eye. Therefore, frequent instillation of eye drops is necessary to maintain a therapeutic drug level in the tear film or at the site of action. But the frequent use of highly concentrated solutions may induce toxic side effects and cellular damage at the ocular surface. It is one of the reasons of non-compliance and failure of therapy. To enhance the amount of active substance reaching the target tissue to exert a local effect in the *cul de sac*, the residence time of the drug in the tear film should be lengthened [2].

Different strategies have been developed to increase the drug bioavailability by prolonging the contact time of the formulation with the corneal / conjunctival epithelium. One of the strategies is to develop ocular mucoadhesive systems to increase the residence time of the drug in the *cul de sac*. Different natural and synthetic mucoadhesive polymers interact with the precorneal mucin layer and show good potential to increase the bioavailability by increasing the precorneal residence time of the drug [2]. This will help to reduce the frequency of administration of the drug dose as well as improve patient compliance.

The eye, being a highly vascular organ, is commonly involved in systemic infections as well as inflammation. It is also affected in diseases of surrounding structures such as the paranasal sinuses and lachrymal sac. Being well-connected to the brain, eye infections can spread to the meninges, venous sinuses, and the brain tissue [3]. The most common causes of ocular inflammation are allergic or infectious in origin [4]. Keratitis is a common infectious disease of the cornea that may even lead to loss of vision. Common risk factors for keratitis are the use of contact lens, diseases of the ocular surface, ocular trauma, and ocular surgery. Common causative organisms associated with ocular keratitis are *Pseudomonas aeruginosa, Staphylococcus aureus, coagulase-negative Staphylococci,* and *Streptococcus pneumoniae*[5, 6]. Of these, *S. aureus* is the predominant pathogen isolated from the majority of keratitis cases, but *P. aeruginosa*, a potentially devastating ocular pathogen, is the most common cause of corneal ulcers, ulcerative keratitis associated with contact lens wear, and severe necrotic corneal ulceration [2, 5].

Moxifloxacin HCl is a fourth-generation fluoroquinolone, having broad-spectrum antibiotic activity, with efficacy against various Gram-positive and Gram-negative microorganisms [5–7] including *Staphylococci, S. pneumoniae*, members of the family *Enterobacteriaceae, P. aeruginosa*, *H. influenza,* and *Moraxella* species, through the inhibition of DNA gyrase and topoisomerase IV. It is commonly used to treat ocular infections and is a pre- and post-operative prophylactic agent in intraocular surgery to prevent endophthalmitis [7]. It has superior corneal and aqueous penetration ability leading to higher therapeutic levels, more effective antimicrobial activity, and better clinical outcomes [8]. Studies revealed that it has improved activity against Gram-positive, atypical, and Gram-negative organisms compared to second- and third-generation fluoroquinolones (i.e. ofloxacin, ciprofloxacin, levofloxacin). It is available as drops (0.5% w/v) for ophthalmic use, and its FDA-approved dosing regimen for the treatment of acute bacterial conjunctivitis is one drop twice a day for seven days [9]. In these formulations, the dosing frequency is quite high. To reduce the dosing frequency and to increase its precorneal residence time, an ocular mucoadhesive system is required. Literature was reviewed for Moxifloxacin HCl ocular dosage forms; only *in situ* hydrogel systems [10, 11] were prepared previously. In this study, a successful attempt was made to formulate and evaluate more efficacious ocular mucoadhesive microspheres of Moxifloxacin HCl.

## Materials and method

### Materials

Moxifloxacin hydrochloride was generously gifted by Orex Pharma Pvt. Ltd. Dombivali, India. Methocel K15M CR Premium and K100M CR Premium were provided by Lupin Pharma Ltd., Pune, India and Colorcon Asia Pvt. Ltd., Verna, Goa, India respectively. Sodium carboxymethylcellulose 7MF and HV were provided by Piramal Healthcare Pvt. Ltd., Mumbai, India and Dupen Laboratories Pvt. Ltd., Mumbai, India respectively. All other chemicals were of analytical grade and used as received.

### FT-IR study

FT-IR spectroscopy was carried out to check the compatibility between the drug and polymer. The FT-IR spectra of the drug with polymers was obtained by the diffused reflectance method using KBr and compared with the standard FT-IR spectra of the pure drug.

### Experimental design

Eight different types of formulations F1, F2, F3, F4, F5, F6, F7, and F8 were prepared, using two different polymers with two different grades and different drug: polymer ratios (1:1 and 1:2).

### Method of preparation of mucoadhesive microspheres

Moxifloxacin-loaded microspheres were prepared by the solvent evaporation heat cross-linking technique. The polymer, polymer grades, quantity of the polymer, and quantity of the drug used for the preparation of each formulation is indicated in Tab. 1. Microspheres were prepared by dissolving a specific quantity of polymer in sufficient distilled water to produce a 2% polymeric solution, and a specific quantity of drug was mixed with the aqueous polymeric solution. This solution was added dropwise to light liquid paraffin containing 0.5% span 80 as an emulsifying agent, with constant stirring at 2000–2500 rpm using a three blade propeller to form a w/o emulsion. The content in the beaker was heated at 50 °C with constant stirring for 5 hours. After complete evaporation of the aqueous phase, the liquid paraffin was decanted, and the collected microspheres were washed three times with n-hexane to remove the liquid paraffin. The microspheres were dried and stored. Each formulation was run in triplicate

### Evaluation of mucoadhesive microspheres

#### Production yield

The obtained Moxifloxacin HCl mucoadhesive microspheres were collected and weighed to determine the production yield (PY) using the following equation 1.

Eq. 1 $$\text{PY}(\%)={\text{W}}_{1}/{\text{W}}_{2}\times 100$$

Where, W_1_ is the weight of dried microspheres and W_2_ is the sum of the initial dry weight of the drug and polymer.

#### Particle size

The particle size of the microspheres was determined by an optical microscope fitted with an ocular micrometer. The ocular micrometer was calibrated previously with the stage micrometer. The microspheres were mounted in liquid paraffin and a diameter of 100 microspheres was measured randomly by the optical microscope.

#### Surface morphology

The surface morphology of the microspheres was visualized by scanning electron microscopy (JEOL JSM-6360 SEM). The samples were prepared by lightly sprinkling the microspheres on a double-sided adhesive tape which was already stuck on aluminum stubs. The stubs were then placed into an ion sputter coater (JEOL JFC-1600) and coated with platinum in the inert environment of argon. After coating, the samples were randomly scanned by an electron beam to get three dimensional images of the microspheres.

#### Actual drug content and encapsulation efficiency

Ten mg of microspheres were accurately weighed and transferred into a 50 ml volumetric flask, the volume was made with simulated tear fluid (STF) pH 7.4, and the microspheres were dissolved by ultra-sonication for 3 h at 25 °C. The STF used was freshly prepared (0.0238 mol/ml NaHCO_3_, 0.1146 mol/ml NaCl and 0.0005 mol/ml CaCl_2_. 2H_2_O in water) [10]. The samples were filtered through a 0.2 μm membrane filter. Five ml of the sample solution was diluted to 50 ml with the same medium and the absorbance of the samples was measured at 288 nm using a UV-spectrophotometer. Actual drug content (AC) and encapsulation efficiency (EE) were calculated using equation 2 and 3 respectively. All analyses were carried out in triplicate.

Eq. 2 $$\text{AC}(\%)={\text{M}}_{\text{act}}/{\text{M}}_{\text{ms}}\times 100$$

Eq. 3 $$\text{EE}(\%)={\text{M}}_{\text{act}}/{\text{M}}_{\text{the}}\times 100$$

Where, M_act_= Actual Moxifloxacin HCl content in microspheres, M_ms_= Weighed quantity of microspheres, M_the_= Theoretical quantity of Moxifloxacin HCl in microspheres calculated from the quantity added in the process.

#### Mucoadhesion test

The mucoadhesion property of the microspheres formulation was determined by modifying the previously described *in vitro* wash-off test for mucoadhesion by *Vyas et al*[12]. The eyeballs along with the eyelids of a freshly sacrificed goat were obtained from a local slaughterhouse within one hour of killing the animal, and was cleaned by washing with isotonic saline solution. A piece of conjunctiva (2 cm×2 cm) and cornea were isolated carefully and mounted onto glass slides using double-sided adhesive tape and thread. An accurate weight of microspheres (5 mg) was spread onto each wet, rinsed tissue specimen and allowed to hydrate for 5 minutes, thereafter the glass slides were immediately hung onto the arms of the USP Disintegration Apparatus with the help of a thread. By operating the Disintegration Test machine, the tissue specimen was given a regular up and down movement in 500 ml of STF pH 7.4 at 37 °C. The tissue specimen was observed microscopically after the specific time interval of 15 min. The time required for detaching all the microspheres from the mucosal surface of the tissue was recorded by visual inspection. The analysis was carried out in triplicate.

#### In vitro release study

The *in vitro* release profiles of Moxifloxacin HCl mucoadhesive microspheres were examined in STF pH 7.4. A ten mg equivalent of Moxifloxacin HCl-loaded mucoadhesive microspheres were placed in separate Eppendorf tubes and 1.5 ml of STF pH 7.4 was added into each tube. The tubes were shaken at 100±5 rpm in a thermostat-controlled orbital shaking incubator at 37±1 °C. At scheduled time intervals of 1 h, the tubes were centrifuged at optimum conditions of 4000 rpm for 1 minute. Optimum centrifugation speed and time, which could separate the supernatant without causing microsphere aggregation, were determined by preliminary studies. Ten μl samples from the supernatant were withdrawn and replaced with fresh medium. The samples were diluted to 5 ml with STF pH 7.4 and absorbance was measured at 288 nm using a UV-spectrophotometer. The study was carried out for 12 h. The analysis was carried out in triplicate.

#### Release kinetics

The mechanism of the drug release was determined by fitting *in vitro* release data to various curve fitting models such as the zero order, first order, Higuchi, Korsemeyer-Peppas, and Hixson Crowell model and finding r-values for the release profile corresponding to each model.

#### Moxifloxacin HCl activity against bacterial strains

The microbiological studies were carried out to ascertain the antimicrobial activity of the prepared formulations and to compare with the marketed eye drop, against *P. aeruginosa* (ATCC 6580) [13] and *S. aureus* (NCTC 6749) [14]. A subculture of each organism was prepared by transferring a loop full of each organism from laboratory maintained cultures into 100 ml of sterilized nutrient broth and incubated for 24 h at 37 °C temperature. Müller-Hinton-Agar medium was inoculated with the subculture (20 ml subculture/100 ml of Müller-Hinton-Agar), and 40 ml of the inoculated medium was transferred to each petri plate and allowed to solidify. Three wells were prepared aseptically in each plate with the help of a stainless steel borer (8 mm diameter) so that the wells were separated equally from each other. The weighed quantities of all microspheres were taken and suspended separately in normal saline solution (0.5% w/v) prior to the transfer into wells. Then 100 μl of each of the test solutions, as well as the marketed eye drops were placed in separate petri plate bores under aseptic conditions. A positive control (petri plate with micro-organism but placed in normal saline) and a negative control (petri plate without microorganism) were also prepared. The results obtained were analyzed statistically by the analysis of variance followed by Dunnette’s Multiple Comparison test using GraphPad Prism Software.

#### Ocular Irritancy Test

The promising ocular mucoadhesive microsphere formulation was subjected to the Draize irritancy test as per OECD test guidelines [15]. The ocular irritation study was performed on male albino rabbits weighing 1–2 kg. Approval by the Institutional Animal Ethics Committee of KLE University’s College of Pharmacy, Belgaum was obtained prior to commencing the study. Six albino rabbits of either sex were used for this study. They were housed and maintained in the animal house at room temperature during the period of the study. They were fed with standard diet and water throughout the experiment.

The animals were divided into three groups viz. positive control, negative control, and test, each containing two animals. The positive control group received 1% w/w solution of dioctyl sodium sulfosuccinate (ocular irritant), the negative control group received normal saline solution, and the test group received eye drops of the promising formulation in light liquid paraffin. One drop of the sterile solutions was instilled into the lower *cul de sac* twice a day for a period of seven days. Rabbits were observed periodically for redness, swelling, and watering of the eyes at the time intervals of 1 h, 24 h, 48 h, and 1 week after administration. Each item was graded using a severity scale of 0 to 3 (0= absent, 1= mild, 2= moderate, and 3= severe) [16].

#### In vivo efficacy testing

Eighteen albino rabbits weighing 1–2 kg of either sex were used for the animal studies. All experiments were conducted according to the “Guide for the Care and Use of Laboratory Animals” [17]. The animals were housed in standard cages and kept in a light-controlled room at 19±1 °C and 50±5% relative humidity without any restriction of food or water. The protocol was approved by the Institutional Animal Ethics Committee of KLE University’s College of Pharmacy, Belgaum. A sterile suspension of the promising formulation in light liquid paraffin and the marketed eye drop, both containing 0.5% w/v of Moxifloxacin HCl were tested for *in vivo* evaluation. Light liquid paraffin was selected as the vehicle to maintain the integrity of particles in the formulation before *in vivo* application, and the formulation was sterilized by autoclaving at 121 °C, 15 psi pressure for 15 min.

*S. aureus* and *P. aeruginosa,* which were proven to be sensitive to Moxifloxacin HCl by preliminary studies, were chosen for *in vivo* experiments and were diluted in sterile normal saline, to induce corneal infection. Eighteen rabbits were divided into three groups, each containing six animals. The first and second groups were infected with microorganisms and the third group was used as the control. The right eye of the rabbits was infected with *P. aeruginosa* (n=6) and left one with *S. aureus* (n=6). For this purpose, the corneas of each rabbit were intrastromally injected with 0.1 ml of the bacterial suspension using a 30-gauge needle after local anesthesia with Proparacaine HCl.

After 6 h of inoculation, bacterial keratitis was confirmed and topical therapy was started. The first group was treated with the marketed eye drop while the second group was treated with the promising formulation eye drops. Treatment consisted of two drops in each eye every 12 h for six days (total 12 doses). The eyes of each animal were observed visually every day throughout the duration of study for symptoms of bacterial keratitis (blepharitis, iritis, conjunctivitis, corneal edema, and corneal infiltrates). Each item was graded using a severity scale of 0 to 3 (0= absent, 1= mild, 2= moderate, and 3= severe). All observations and grading were done by an ophthalmologist. Treatment effects were compared with those of the marketed formulations, and the significance was determined by the following t-test. Significance levels (P<0.05) were determined by a two tailed t-test. Significance values obtained from the treatment were compared with the theoretical t-value. Treatment was found to be significant (S) if the t-value exceeded the theoretical t-value. If the treatment t-value did not exceed the theoretical t-value, then the treatment was considered as non-significant (NS) as compared with the marketed eye drops [18].

#### Stability study

Stability testing of pharmaceutical products is done to ensure the efficacy, safety, and quality of active drug substances and dosage forms. As per ICH guidelines, microspheres were subjected to accelerated stability studies. Weighed quantities of the samples (n=3) were kept in glass vials, sealed with rubber plugs, and exposed to controlled temperature (40±2 °C) and relative humidity (75±5%) for a period of three months in a humidity control oven (Lab Control, Ajinkya IM 3500 Series, India). After 30, 60, and 90 days, the samples were taken out and analyzed for appearance, particle size, entrapment efficiency, and *in vitro* release. A 90 days stability testing was also evaluated by Differential Scanning Calorimetry.

## Results and Discussion

Ocular mucoadhesive microspheres of Moxifloxacin HCl were prepared by using Methocel and Sodium CMC as mucoadhesive polymers. The effects of viscosity and concentration on different parameters were studied by preparing eight different formulations with different viscosity polymers and varying drug: polymer ratios.

### FT-IR study

**Fig. 1:**
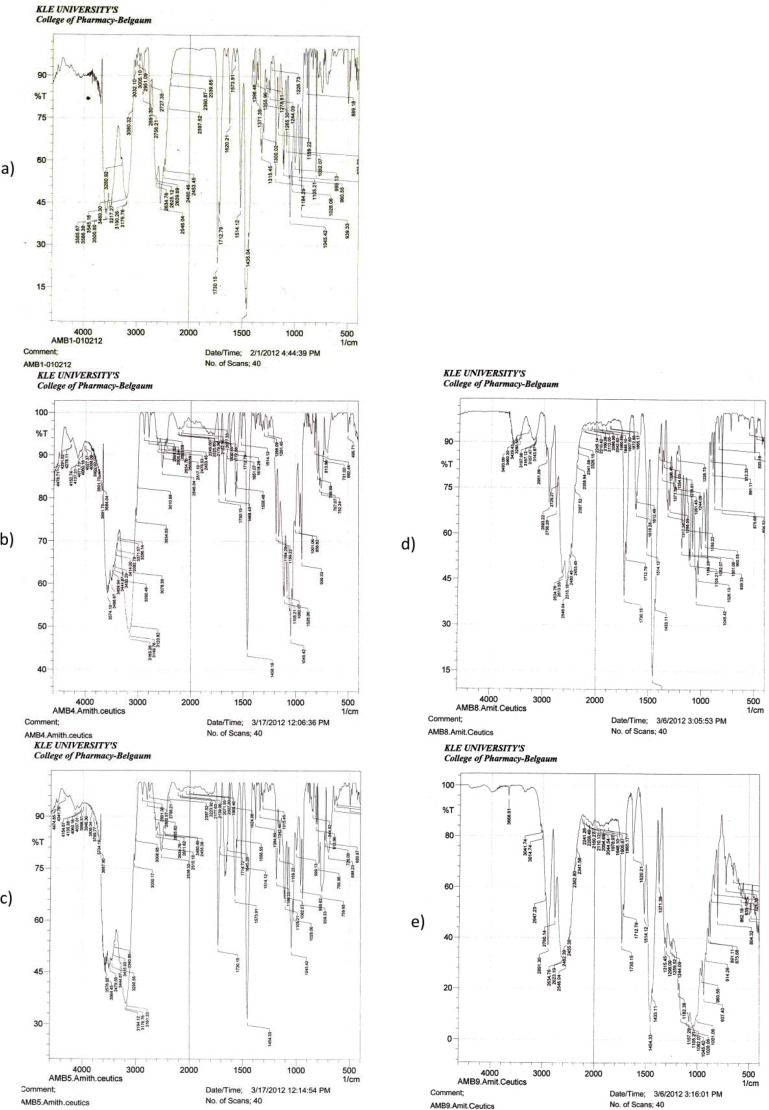
FT-IR Spectra a: Moxifloxacin HCl b: Moxifloxacin HCl with Methocel K15M c: Moxifloxacin HCl with Methocel K100M d: Moxifloxacin HCl with Sodium CMC 7MF e: Moxifloxacin HCl with Sodium CMC HV)

Preformulation studies were carried out prior to the preparation of mucoadhesive microspheres to study the compatibility of the pure drug Moxifloxacin HCl with the polymers Methocel K15M, Methocel K100M, Sodium CMC 7MF, and Sodium CMC HV. The individual IR spectra of the pure drug as well as the combination spectra of the drug and polymers are shown in the Fig. 1 (Tab. 2). All important functional group frequencies for Moxifloxacin HCl [19] were also present in the combination spectra which indicate no interaction between Moxifloxacin HCl and polymers.

### Practical yield

The practical yield of different formulations is given in Tab. 3. The practical yield was found to be the highest for formulation F4 containing high viscosity Methocel K100M in the drug: polymer ratio of 1:2. The practical yield was found to be increased with increasing viscosity as well as concentration of the polymer. This is because a greater amount of polymer was added in the same volume of the continuous phase [20].

### Particle size

The particle sizes of different microspheres prepared using Methocel (F1–F4) were found to be in the range of 29.10±14.97 μm to 57.60±21.92 μm, while those prepared with Sodium CMC (F5-F8) were found to be in the range of 31.65±12.64 μm to 78.45±25.84 μm as shown in Tab.3. In the case of Methocel microspheres, results showed that as the viscosity and concentration of polymers increased, particle size decreased. This may be due to the increase in the availability of the polymer for the entrapment of drug particles improving cross-linking [21]. In the case of microspheres prepared using Sodium CMC, the size of particles was increased with an increase in viscosity of polymers, but was reduced with an increase in the concentration of polymers. An increase in particle size with respect to viscosity may be due to an increase in droplet size during the addition of polymer solution in the oily phase [22]. Meanwhile, a decrease in particle size with respect to concentration may be due to the increase in availability of a greater amount of polymer for the entrapment of the drug particle [21].

### Surface morphology

**Fig. 2:**
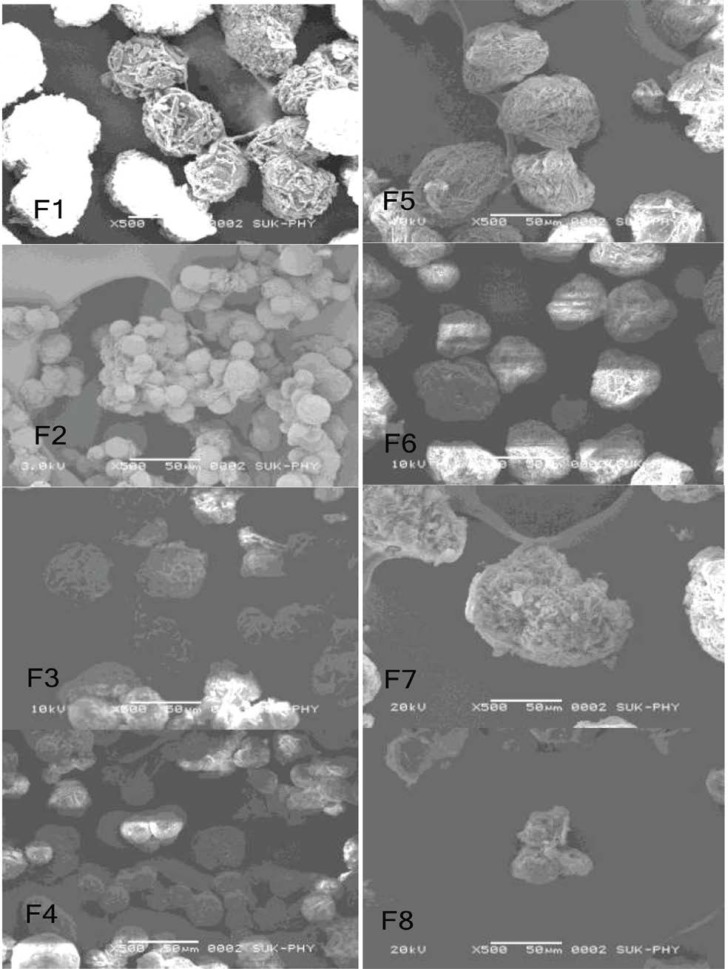
SEM Images of Formulation F1 to F8. (X500)

The surface morphology of the prepared microspheres was examined by scanning electron microscopy (SEM). All microspheres were found to be spherically shaped when observed with an optical microscope, but when observed by SEM, microspheres prepared with Methocel were found to be more spherical and smooth than those prepared with Sodium CMC (Fig. 2). The surface morphology of Methocel microspheres (F1 to F4) was dependent on the concentration of polymers. Methocel microspheres having a higher proportion of polymers were found to have a smooth surface due to the availability of more polymers for cross-linking causing the entrapment of more drug. The shape of the Sodium CMC microspheres was found to be distorted, having a rough and fractured surface, as sodium CMC is more water-soluble than Methocel at high temperature [23], and the hydrophilicity of Sodium CMC microspheres became stronger at the cross-linking temperature which interfered with proper cross-linking of polymer. This may be the reason behind the distorted shape and rough surface of Sodium CMC microspheres [24]. At high concentration of the polymers (F2, F4, F8), some of the microspheres were fused to each other, which may be due to the presence of higher amount of water, which slowly evaporates upon stirring, causing the particles to come in contact with each other [25].

### Actual drug content and entrapment efficiency

The actual drug content was found to be in the range of 24.88±0.24% to 50.75±0.40% (Tab. 3). It was reduced with respect to the concentration of the polymer used, as the same quantity of drug was added in a higher quantity of the polymer. Entrapment efficiency was found to be minimum for formulation F1 (76.90±1.56%) and maximum for formulation F7 (101.49±0.81). Entrapment efficiency of all formulations is given in Tab. 3. It was improved with an increase in polymer concentration because a higher quantity of polymer was available for cross linking which prevented drug diffusion. Also, Moxifloxacin HCl is sparingly water-soluble which restricts itself in the aqueous phase [22].

### Mucoadhesion Test

Ocular mucoadhesion of microspheres was the most important aspect of the present work. It was investigated by modifying the *in vitro* wash-off test described by Vyas *et al*[12], using the conjunctiva and cornea of a goat. Corneal mucoadhesion of prepared microspheres was found to be 3.53±0.20 h to 8.45±0.40 h and conjunctival mucoadhesion was found to be 4.35±0.17 h to 9.40±0.53 h (Tab. 3). Results showed that conjunctival mucoadhesion was better than corneal mucoadhesion, as the conjunctival epithelium is thicker than the corneal epithelium and possesses mucus-secreting goblet cells [26]. It was found that mucoadhesion time was dependent on both the viscosity as well as concentration of polymers. An increase in viscosity and concentration of polymers improves mucoadhesion time. Results showed that Methocel was a better ocular mucoadhesive polymer than Sodium CMC. Swelling and expansion of the polymer chain for interpenetration and entanglement of the polymer with the mucous network is considered to be responsible for mucoadhesion [27]. Formulation F4, containing Methocel K100M (1:2), exerted longer mucoadhesion time and was selected for further *in vivo* evaluation.

### In vitro release

**Fig. 3:**
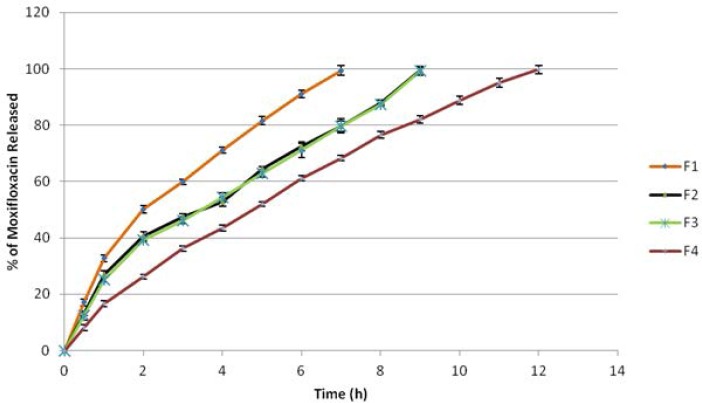
Comparative Drug release profile of Moxifloxacin HCl from Ocular Mucoadhesive Microsphere formulations F1 to F4. Values are the mean of three runs.

**Fig. 4:**
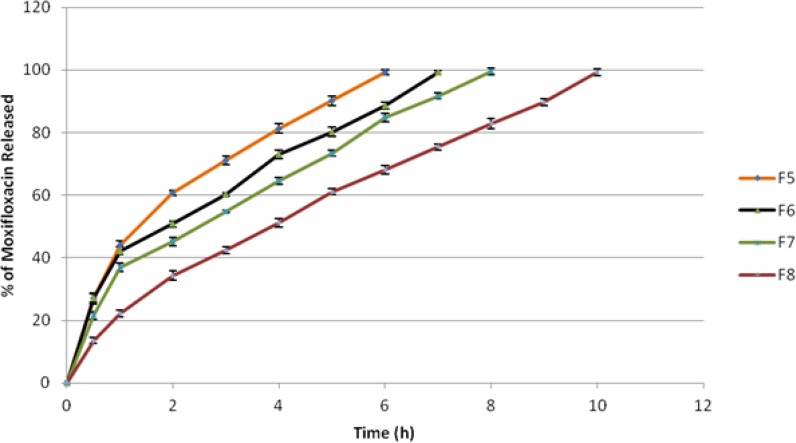
Comparative drug release profile of Moxifloxacin HCl from ocular mucoadhesive microsphere formulations F5 to F8. Values are the mean of three runs.

The cumulative percentage release of Moxifloxacin HCl from all mucoadhesive microspheres is shown in Fig. 3 and 4. The effect of type, viscosity, and concentration of polymers on drug release was studied. Formulation F1 and F2 containing 500 mg and 1000 mg of Methocel K15M respectively, released 99.40% and 98.55% at the end of 7 h and 9 h respectively. Formulation F3 and F4 containing 500 mg and 1000 mg of Methocel K100M respectively, released 99.39% and 99.71% at the end of 9 h and 12 h respectively. Formulation F5 and F6 containing 500 mg and 1000 mg of Sodium CMC 7MF respectively, released 99.37% and 99.24% at the end of 6 h and 7 h respectively. Formulation F7 and F8 containing 500 mg and 1000 mg of Sodium CMC HV respectively, released 99.57% and 99.31% at the end of 8 h and 10 h respectively. Thus, it was clearly evident that drug release decreased with an increase in viscosity as well as concentration of polymers used, owing to more polymer entanglement and greater gel strength. The order of microspheres showing an increasing release rate was F4<F8<F2<F3<F7<F6<F1<F5. Sodium CMC microspheres gave a relatively fast release as compared to Methocel microspheres, due to rapid swelling and rapid dissolution of Sodium CMC in the dissolution medium [28]. Also, the hydration rate of Methocel is higher and it forms a strong viscous gel when in contact with aqueous media which may retard the drug release [29].

### Release kinetics

In order to know the mechanism of drug release from ocular mucoadhesive microspheres of Moxifloxacin HCl, data of *in vitro* release studies were extrapolated by the zero order, first order, Higuchi’s, Korsmeyer-Peppas, and Hixson-Crowell equations. The applicability of all of these equations was tested and summarized in Tab. 4. The rate constants were also calculated from the slope of the plot of the respective models. From the dissolution data of all of the formulations when fitted in accordance with Higuchi’s square root equation, a linear relationship was obtained with an ‘r’ (correlation coefficient) value close to unity (0.9864 to 0.9976) and higher than ‘r’ obtained from the zero order equation (0.9587 to 0.9896), the first order equation (0.6787 to 0.7974), and the Hixen Crowell equation (0.8761 to 0.9435). To find out the exact mechanism, dissolution data of all formulations were fitted in the Korsmeyer-Peppas equation. All formulations showed good linearity (r: 0.9833 to 0.9987), with slope (n) values ranging from 0.4642 to 0.7709. The zero order rate equation describes the system, where release rate is independent of the concentration of the dissolved species [30]. The first order equation describes the release from the systems where dissolution rate is dependent on the concentration of the dissolving species [31]. The rate laws predicted by the different mechanisms of dissolution both alone and in combination, have been discussed by Higuchi [32]. The Hixen-Crowell equation describes the drug release from the pharmaceutical dosage form by the erosion mechanism. The Korsmeyer-Peppas equation is used to analyze the release of pharmaceutical polymeric dosage forms, when the release mechanism is not well-known or when more than one type of release phenomena could be involved [31]. All formulations were best fitted in the Higuchi equation, indicating diffusion to be the predominant mechanism of drug release [30].

To find out the exact mechanism, dissolution data of all of the formulations were fitted in the Korsmeyer-Peppas equation. In the Korsmeyer-Peppas model, ‘n’ is the release exponent indicative of the mechanism of drug release. A value of n = 0.45 indicates Fickian or case I release; 0.45 < n < 0.89 indicates non-Fickian or anomalous release; n = 0.89 indicates case II release; and n > 0.89 indicates super case II release. In the Fickian release mechanism, the rate of drug release is much less than that of polymer relaxation (erosion), so the drug release is chiefly dependent on the diffusion through the matrix. In the non-Fickian (anomalous) case, the rate of drug release is due to the combined effect of drug diffusion and polymer relaxation. Case II release generally refers to the polymer relaxation [33]. The ‘n’ values for all formulations were between 0.45 to 0.89, indicating that the release mechanism was non-Fickian or anomalous release. Thus, drug release from mucoadhesive microspheres was controlled mainly by diffusion as well as polymer relaxation.

### Moxifloxacin HCl activity against bacterial strains

All ocular mucoadhesive microsphere formulations and marketed conventional eye drops of Moxifloxacin HCl were evaluated for antimicrobial activity by the cup-plate method. All formulations gave a clear zone of inhibition comparable with marketed eye drops (Tab. 5). The diameter of the zone of inhibition was 48.67±2.31 mm and 51.33±1.15 mm after 24 h for the marketed eye drop and formulation F4 respectively. Formulation F4 showed a diameter of the zone of inhibition to be greater than that of the marketed formulation against test organisms, as the drug slowly diffuses from the microspheres for a longer duration of time, preventing growth of microorganisms. The results obtained were compared statistically with the negative control (without drug) by One-way ANOVA, followed by Dunnett’s Multiple Comparison Test using GraphPad Prism software. The comparison of all formulations with the control group by Dunnett’s multiple comparison test revealed that the results obtained by all formulations and the marketed eye drops were statistically significant as P<0.05 for all of the formulations and marketed eye drops.

### Ocular irritancy test

Ocular mucoadhesive microsphere formulation F4 showed no signs of redness, watering, and swelling of the eyes throughout the ocular irritancy study as given in Tab. 6. Formulation F4 showed excellent ocular tolerance.

### In vivo efficacy testing

*In vivo* efficacy in bacterial keratitis induced by *P. aeruginosa* and *S. aureus* of the promising formulation F4 was compared with the conventional marketed eye drops both containing 0.5% w/v of Moxifloxacin HCl. Bacterial keratitis was induced by intrastromally injecting corneas with the bacterial suspension. The right eyes of rabbits were infected with *P. aeruginosa* and left eyes with *S. aureus*. The first group was treated with the marketed eye drops of Moxifloxacin HCl, while the second group was treated with the formulation F4 microspheres suspension in light liquid paraffin. The eyes were observed for blepharitis, iritis, conjunctivitis, corneal edema, and corneal infiltration on the score basis for six days by an ophthalmologist.

An improvement in the symptoms was observed for ocular mucoadhesive microsphere formulation F4. It was found that *S. aureus* was more sensitive to Moxifloxacin HCl than *P. aeruginosa* as eyes infected with *S. aureus* (Left) recovered quicker than those infected with *P. aeruginosa* (right). Results showed (Tab. 7) that bacterial keratitis induced by *S. aureus* was cured on the 3rd and 5th day with formulation F4 and the marketed eye drops respectively. In the case of *P. aeruginosa*, it was found that it produced a severe form of keratitis which led to severe conjunctivitis and corneal infiltrates, and it was cured on the 4th and 6th day with formulation F4 and the marketed eye drops respectively. Thus, the bacterial keratitis induced in rabbits was successfully treated with ocular mucoadhesive microsphere formulation F4 in a much shorter time compared with the conventional marketed eye drops. Due to the mucoadhesive nature of microspheres, its retention time in the eye was improved, avoiding the loss of drug through nasolacrimal drainage. The drug was released slowly and was available for a longer duration at the site, hence, improving the symptoms within a shorter period of time.

The results were analyzed statistically by applying the two-tailed t-test (P<0.05) and the results were compared with the theoretical value (2.228) obtained from t-table. Treatment was significant if the calculated t-value exceeded the theoretical t-value. Statistical data is given in Tab. 8, which shows that significant changes were observed earlier in the case of formulation F4 as compared with the marketed eye drops.

### Stability study

**Fig.5:**
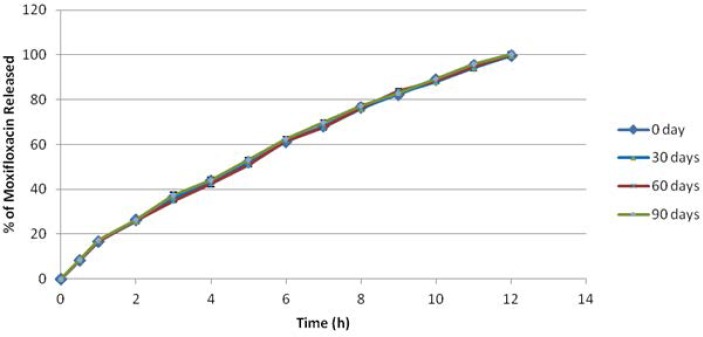
Comparative drug release data of Formulation F4 at 0, 30, 60, and 90 days of stability study. Values are the mean of three runs.

**Fig. 6:**
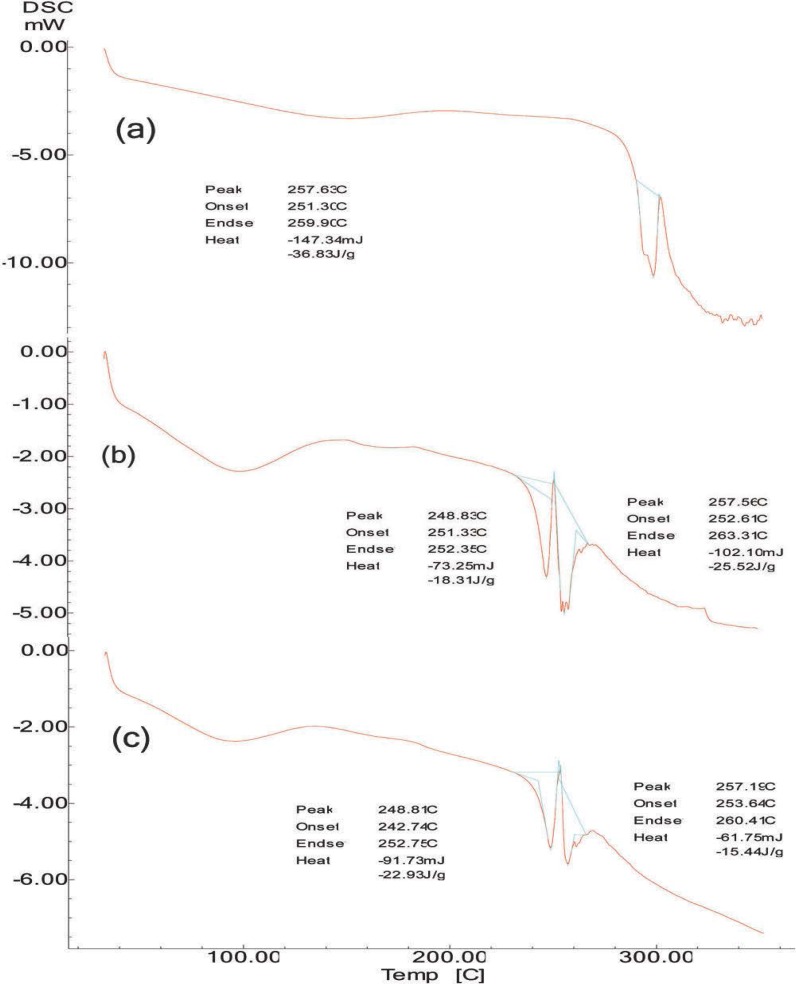
DSC thermo grams. a: Moxifloxacin HCl b: Formulation F4 before stability study c: Formulation F4 after 90 days of stability study

Results showed that there were no significant changes observed in the appearance, particle size, entrapment efficiency (Tab. 9), and *in vitro* release analysis of the formulation (Fig. 5). DSC thermograms of the pure drug and formulation F4, before and after the stability test (Fig. 6), showed a sharp endothermic peak at 257 °C of Moxifloxacin HCl, and thermograms of the formulation showed another sharp endothermic peak at 248 °C of Methocel, indicating that the microspheres were stable after 90 days. It thus confirmed that formulation F4 was stable at the end of 90 days.

## Conclusion

The formulation of ocular mucoadhesive microspheres appears to be suitable for the ocular application and showed excellent ocular tolerance. The prepared ocular mucoadhesive microspheres of Moxifloxacin HCl, due to their ocular mucoadhesive property, improved pre-corneal residence time of the drug, thereby decreasing the total duration of the therapy against bacterial keratitis when compared with conventional eye drops. The efficacy of Moxifloxacin HCl in the treatment of bacterial keratitis was improved by formulating its ocular mucoadhesive microspheres.

## Figures and Tables

**Tab. 1.** Composition of formulations

**Formulation**	**MOX (g)**	**K15M (g)**	**K100M (g)**	**SCMC 7MF (g)**	**SCMC HV (g)**	**Span 80 (%)**	**Light Liquid Paraffin (ml)**
F1	0.5	0.5	–	–	–	0.5	100
F2	0.5	1.0	–	–	–	0.5	100
F3	0.5	–	0.5	–	–	0.5	100
F4	0.5	–	1.0	–	–	0.5	100
F5	0.5	–	–	0.5	–	0.5	100
F6	0.5	–	–	1.0	–	0.5	100
F7	0.5	–	–	–	0.5	0.5	100
F8	0.5	–	–	–	1.0	0.5	100

a MOX-Moxifloxacin HCl, K15M-Methocel K15M, K100M-Methocel K100M, SCMC 7HV-Sodium CMC 7MF, SCMC HV-Sodium CMC HV

**Tab. 2.** Comparison of FT-IR spectral peaks of pure drug and drug with polymers

**Functional Group**	**Reported Peaks^a^ (cm^−1^)**	**Observed Peaks (cm^−1^)**				
		**MOX**	**MOX + K15M**	**MOX + K100M**	**MOX + SCMC 7MF**	**MOX + SCMC HV**
-C=O	1623	1620	1618	1624	1618	1620
	1708	1712	1712	1712	1712	1712

-N-H	3496	3496	3496	3495	3493	3493

-OH	3527	3531	3531	3531	3531	3531

Other Important Peaks	2950	2951	2951	2949	2951	2947
	2894	2889	2889	2889	2893	2891
	2456	2455	2455	2455	2453	2455
	1730	1730	1730	1732	1730	1730
	1516	1514	1514	1514	1514	1514
	1456	1454	1454	1454	1454	1454
	1371	1371	1371	1371	1371	1371
	1326	1317	1317	1317	1317	1315
	1185	1184	1184	1186	1184	1182
	1043	1043	1043	1043	1045	1045
	994	993	993	993	1001	1001
	938	937	937	937	939	937
	875	875	875	875	875	875
	835	835	835	835	835	835
	804	804	804	804	804	804

a Standard reference; MOX…Moxifloxacin HCl obtained sample; K15M…Methocel K15M; K100M…Methocel K100M; SCMC 7MF…Sodium CMC 7MF; SCMC HV…Sodium CMC HV.

**Tab. 3.** Parameters evaluated and data obtained for ocular mucoadhesive microspheres.

	**Practical Yield^a^(%)**	**Particle size^a^(μm)**	**Actual Drug Content^a^(%)**	**Entrapment Efficiency^a^(%)**	**Mucoadhesion time^a^(h.min)**	
					**Cornea**	**Conjunctiva**
F1	68.57±2.62	57.60±21.92	38.45±0.78	76.90±1.56	4.00±0.15	4.40±0.08
F2	70.18±2.19	29.10±14.97	24.44±0.31	97.76±1.26	5.10±0.23	5.35±0.23
F3	73.63±1.42	36.15±18.10	46.97±0.66	93.95±1.32	6.35±0.31	6.55±0.23
F4	77.78±2.07	29.18±14.88	24.88±0.24	99.50±0.97	8.45±0.40	9.40±0.53
F5	61.40±1.87	57.60±17.41	46.02±1.13	92.05±2.27	3.53±0.20	4.35±0.17
F6	65.89±1.23	31.65±12.64	24.97±0.60	99.89±2.42	4.55±0.23	5.25±0.31
F7	72.07±1.89	78.45±25.84	50.75±0.40	101.49±0.8	5.45±0.15	5.55±0.08
F8	74.29±1.13	43.95±11.53	25.15±0.38	100.60±1.5	6.50±0.38	7.15±0.15

a mean±s.d. of 3 runs

**Tab. 4.** Model fitting data of release profile for formulations F1 to F8.

	**F1**	**F2**	**F3**	**F4**	**F5**	**F6**	**F7**	**F8**
Zero order (r)	0.9709	0.9821	0.9844	0.9896	0.9587	0.9822	0.9851	0.9914
First order (r)	0.7818	0.7626	0.6787	0.7215	0.7974	0.7493	0.7325	0.7165
Higuchi’s (r)	0.9976	0.9864	0.9874	0.9921	0.9950	0.9890	0.9885	0.9915
Hixen-Crowell (r)	0.9396	0.9106	0.8761	0.9267	0.9435	0.9095	0.9138	0.9047
Korsmeyer- (r)	0.9879	0.9833	0.9868	0.9972	0.9908	0.9868	0.9863	0.9987
Peppas (n)	0.6376	0.6406	0.6663	0.7709	0.5009	0.4642	0.5216	0.6485

r…regression coefficient; n…slope.

**Tab. 5.** Antimicrobial activity of ocular mucoadhesive microspheres and marketed product against *P. aeruginosa* and *S. aureus.*

	**NC**	**F1**	**F2**	**F3**	**F4**	**F5**	**F6**	**F7**	**F8**	**M**
ZOI (mm)^a^	0	48.67± 2.30	49.33± 3.05	46.67± 1.15	51.33± 1.15	47.33± 2.31	46.67± 1.15	48.00± 2.00	46.67± 1.15	48.67± 2.31
Dunnett’s Test (P<0.05)		S	S	S	S	S	S	S	S	S

a mean±s.d. of 3 runs; NC…Negative Control (Without Drug); M…Marketed eye drops; ZOI…diameter of zone of inhibition; S…significant.

**Tab. 6.** Ocular irritancy testing data of ocular mucoadhesive microspheres formulation F4 in rabbit eyes.

	**Scores for Redness^a^**					**Scores for Swelling^a^**					**Scores for Watering^a^**				

	**1 h**	**24 h**	**48 h**	**Day 7**	**Total**	**1 h**	**24 h**	**48 h**	**Day 7**	**Total**	**1 h**	**24 h**	**48 h**	**Day 7**	**Total**
F4	0	0	0	0	0	0	0	0	0	0	0	0	0	0	0
DOS	3	3	3	3	12	2	3	3	3	11	3	3	3	3	12
NS	0	0	0	0	0	0	0	0	0	0	0	0	0	0	0

a mean of 2 runs; F4…formulation; DOS…dioctyl sodium sulfosuccinate; NS…normal saline.

**Tab. 7.** *In vivo* efficacy comparison of ocular mucoadhesive microspheres formulation F4 with conventional marketed eye drops in bacterial keratitis induced by *P. aeruginosa* and *S. aureus.*

**Organism**	**Day**	**B^a^**		**I^a^**		**C^a^**		**CE^a^**		**CI^a^**	

		**M**	**F**	**M**	**F**	**M**	**F**	**M**	**F**	**M**	**F**
*S. aureus* (Left Eye)	1	2.67±0.5	2.17±0.4	2.67±0.5	2.17±0.4	3.00±0.0	2.50±0.5	2.67± 0.5	2.17±0.4	2.33±0.5	2.00±0.0
	2	1.67± 0.5	1.17± 0.4	1.50±0.5	1.17±0.4	2.00±0.0	1.33±0.5	1.33± 0.5	1.17±0.4	1.33± 0.5	1.00± 0.0
	3	0.67± 0.5	0	0.67± 0.52	0	0.83± 0.4	0	0.50± 0.5	0	0.33± 0.5	0
	4	0	0	0.33± 0.5	0	0.50± 0.5	0	0.16± 0.4	0	0	0
	5	0	0	0	0	0	0	0	0	0	0
	6	0	0	0	0	0	0	0	0	0	0

*P. aeruginosa* (Right Eye)	1	3.00±0.0	2.66±0.51	3.00±0.0	2.83±0.4	3.00±0.0	2.83±0.4	3.00±0.0	2.83±0.4	3.00± 0.0	2.83±0.4
	2	2.67±0.5	1.83±0.7	2.83± 0.4	2.33±0.5	2.83±0.4	2.33±0.52	2.83± 0.4	2.33±0.5	2.83± 0.4	2.50±0.5
	3	2.33±0.5	1.17±0.4	2.50± 0.5	1.33±0.5	2.50±0.5	1.17±0.4	2.50± 0.5	1.33±0.5	2.33± 0.5	1.33±0.5
	4	1.63±0.5	0	1.67±0.5	0	1.50±0.5	0	1.67±0.5	0	1.50±0.5	0
	5	0.67±0.5	0	0.67±0.5	0	0.50±0.5	0	0.50±0.5	0	0.50±0.5	0
	6	0	0	0.17± 0.4	0	0.17± 0.4	0	0	0	0.17± 0.4	0

a mean±s.d. of 6 animals; M…Marketed eye drops; F…Formulation F4; B…Blepharitis; I…Iritis; C…conjunctivitis; CE…Corneal edema; CI…Corneal Infiltrate.

**Tab. 8.** *T-*test results data of formulation F4 v/s conventional marketed eye drops.

**Day**	***t* value**									
	***S. aureus* (Left Eye)**					***P. aeruginosa* (Right Eye)**				

	**B**	**I**	**C**	**CE**	**CI**	**B**	**I**	**C**	**CE**	**CI**
1	1.860	1.860	2.236	1.860	1.581	1.581	1.000	1.000	1.000	1.000
	(NS)	(NS)	(S)	(NS)	(NS)	(NS)	(NS)	(NS)	(NS)	(NS)
2	1.860	1.195	3.162	0.620	1.581	2.236	1.860	1.860	1.860	1.195
	(NS)	(NS)	(S)	(NS)	(NS)	(S)	(NS)	(NS)	(NS)	(NS)
3	3.162	3.162	5.000	2.236	1.581	4.341	3.796	4.780	3.796	3.354
	(S)	(S)	(S)	(S)	(NS)	(S)	(S)	(S)	(S)	(S)
4	0	1.581	2.236	1.000	0	7.905	7.905	6.708	7.905	6.708
		(NS)	(S)	(NS)		(S)	(S)	(S)	(S)	(S)
5	0	0	0	0	0	3.162	3.162	2.236	2.236	2.236
						(S)	(S)	(S)	(S)	(S)
6	0	0	0	0	0	0	1.000	1.000	0	1.000
							(NS)	(NS)		(NS)

B…Blepharitis; I…Iritis; C…Conjunctivitis; CE…Corneal edema; CI…Corneal Infiltrates; Theoretical *t* value of *P≤0.05* for two tails=2.228; S…significant (if calculated *t* values are greater than theoretical *t* value); NS…Non-significant.

**Tab. 9.** Stability study data of ocular mucoadhesive microspheres formulation F4.

**Period (Days)**	**Particle size^a^ (μm)**	**Entrapment efficiency^b^ (%)**
00	29.18±14.88	49.75±0.49
30	28.88±14.71	49.49±0.34
60	28.95±14.96	49.30±0.34
90	29.10±14.90	49.27±0.69

a mean±s.d. of 100 particles;

b mean±s.d. of 3 runs.
